# Diagnosis of metaplastic synovial cysts in clinical dermatology

**DOI:** 10.1002/ski2.168

**Published:** 2022-09-14

**Authors:** Paayal S. Vora, Sonam Rama, Stephen Olsen

**Affiliations:** ^1^ Northeast Ohio Medical University Rootstown OH USA; ^2^ Forefront Dermatology Plymouth MI USA; ^3^ Trinity Health IHA Medical Group, Pathology & Laboratory Management Ypsilanti MI USA

## Abstract

Metaplastic synovial cysts are a rare clinical finding, with less than 70 cases diagnosed to date. These cysts are painful and can greatly impact patients' quality of life. The goal of this case report is to describe the manifestations of these cysts to aid in clinical diagnosis and management.

We herein report a case of a 58‐year‐old male who presented with a bump on the lateral left knee. He experienced pain upon walking. Notably, he had a past medical history of left knee replacement 15 years prior to presentation. Upon physical exam, the bump resembled a firm subcutaneous nodule. It was subsequently biopsied via eight‐mm punch excision, revealing a metal portion of the patient's knee replacement; biopsy resulted in the removal of the entire bump. Biopsy results showed a cystic space in the deep dermis containing papillary villous projections consisting of fibrous cores, partially surfaced by a synovial‐like lining. Based on these results, a metaplastic synovial cyst was diagnosed.

The patient was referred to orthopaedic surgery for replacement to prevent recurrence, as the metal in the knee replacement was presumed the source of the cyst. The patient was then reassessed 3 months later, and he described resolution of his knee pain. Physical exam showed a well‐healed linear scar.

This patient's history and exam findings, along with the dermatopathology results, reflect the characteristic pattern in patients suffering from metaplastic synovial cysts. Prompt identification and subsequent removal can significantly improve patient's pain and ability to carry out daily activities.

## Graphical abstract

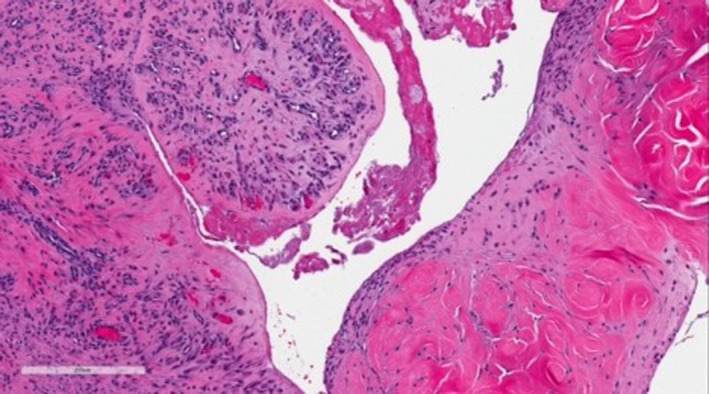

This case report demonstrates a characteristic clinical presentation of Metaplastic Synovial Cysts. The patient presented with a painful subcutaneous nodule 15 years after knee replacement. Dermatopathological analysis of the biopsy specimen demonstrated a cystic space in the deep dermis containing papillary villous projections consisting of fibrous cores, partially surfaced by a synovial‐like lining.

## 1 INTRODUCTION

Metaplastic synovial cysts (MSCs) are a rare clinical finding, with less than 70 cases diagnosed to date. These cysts are painful and can greatly impact patients' quality of life. The goal of this case report is to describe the manifestations of these cysts to aid in clinical diagnosis and management.

## 2 CASE REPORT

The patient was a 58‐year‐old Caucasian male who presented to the clinic with a bump on his left lateral knee. He described pain with movement, especially upon walking. Of note, he had a past medical history of left knee replacement 15 years prior to his presentation.

**FIGURE 1:**
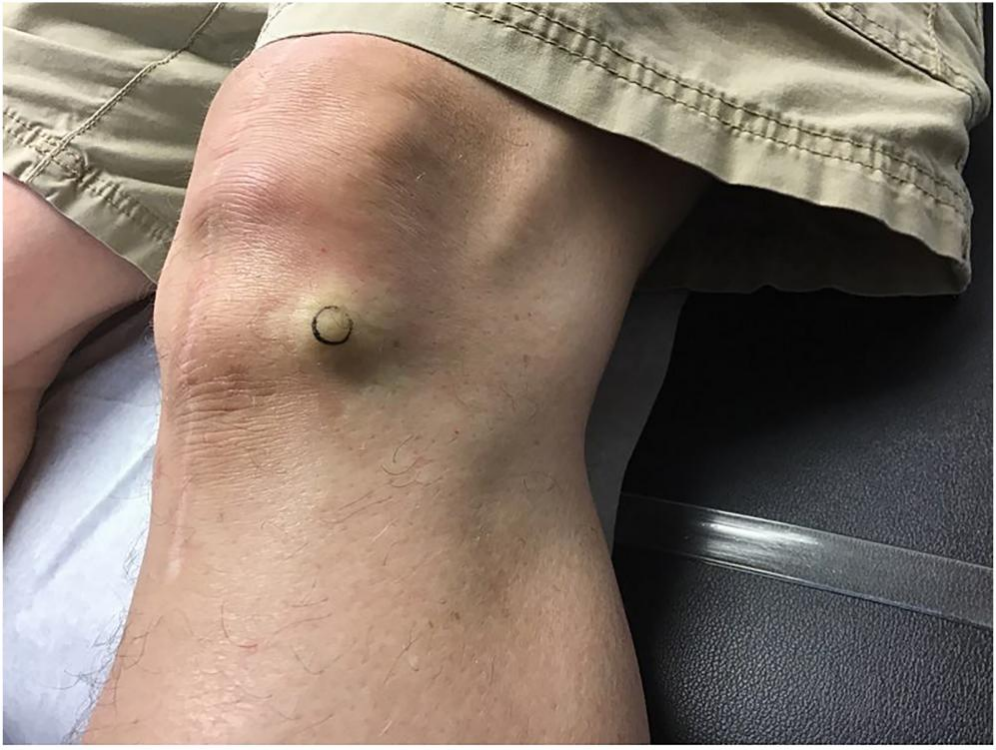
Patient at presentation with bump on left lateral knee, pre‐biopsy

**FIGURE 2:**
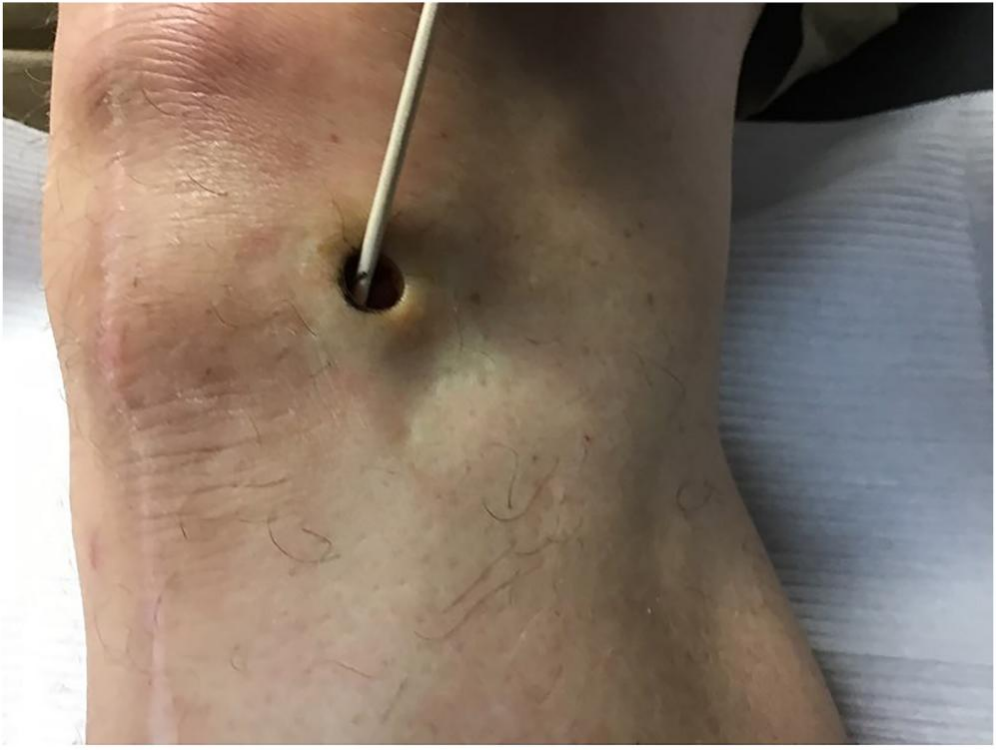
Site of 8‐mm punch biopsy, resulting in removal of entire subcutaneous nodule

**FIGURE 3:**
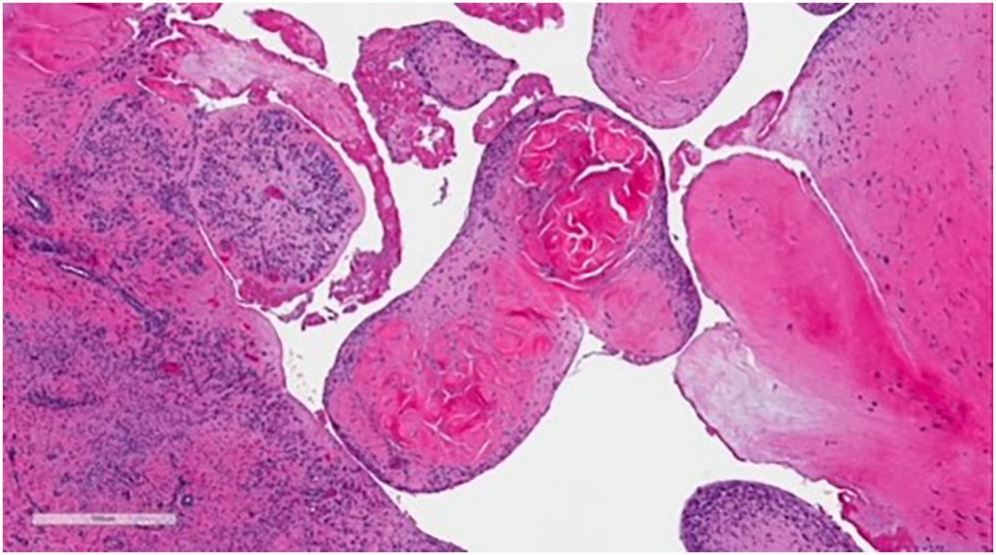
Dermatopathology results of the biopsy specimen, displaying a cystic space in the deep dermis containing papillary villous projections consisting of fibrous cores, partially surfaced by a synovial‐like lining

**FIGURE 4:**
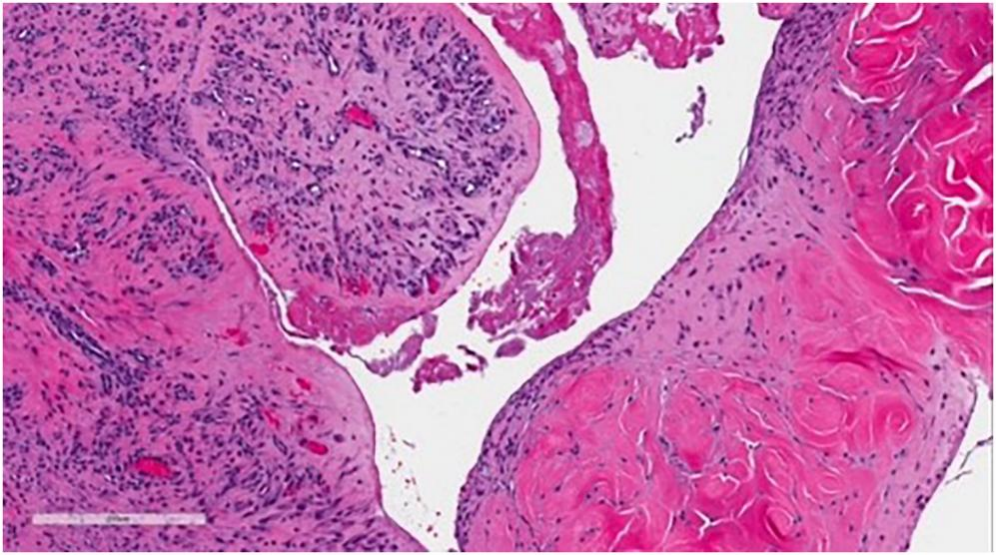
Dermatopathology results of the biopsy specimen, displaying a cystic space in the deep dermis containing papillary villous projections consisting of fibrous cores, partially surfaced by a synovial‐like lining

**FIGURE 5:**
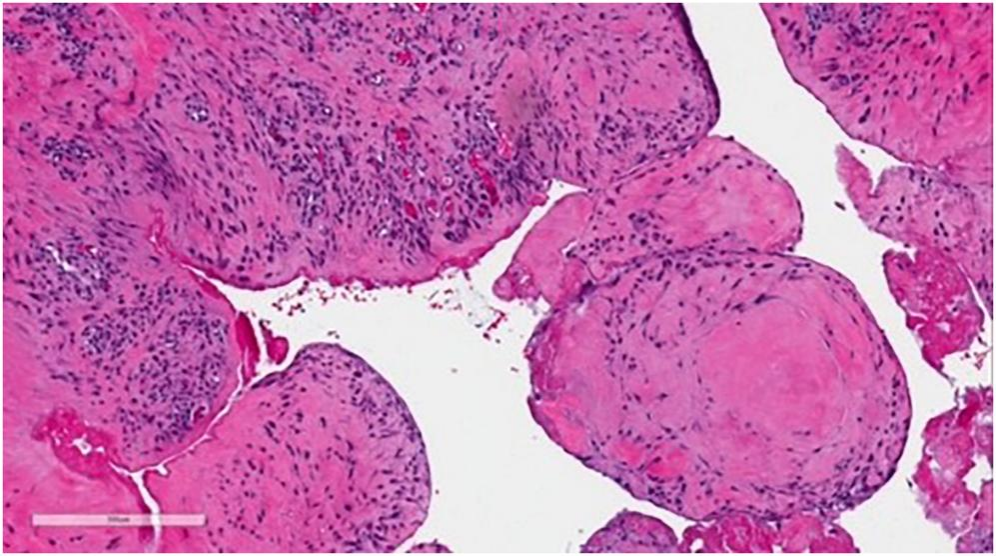
Dermatopathology results of the biopsy specimen, displaying a cystic space in the deep dermis containing papillary villous projections consisting of fibrous cores, partially surfaced by a synovial‐like lining

Upon physical exam, the bump resembled a firm, subcutaneous nodule (Figure 1). An eight‐mm punch biopsy was obtained, resulting in removal of the entire bump (Figure 2). At the base, the biopsy specimen revealed a metal portion of the patient's prior knee replacement. Dermatopathology results detailed a cystic space in the deep dermis containing papillary villous projections consisting of fibrous cores, partially surfaced by a synovial‐like lining ( Figures 3, 4, 5‐3, 4, 5). Based on these results, a metaplastic synovial cyst was diagnosed.

**FIGURE 6:**
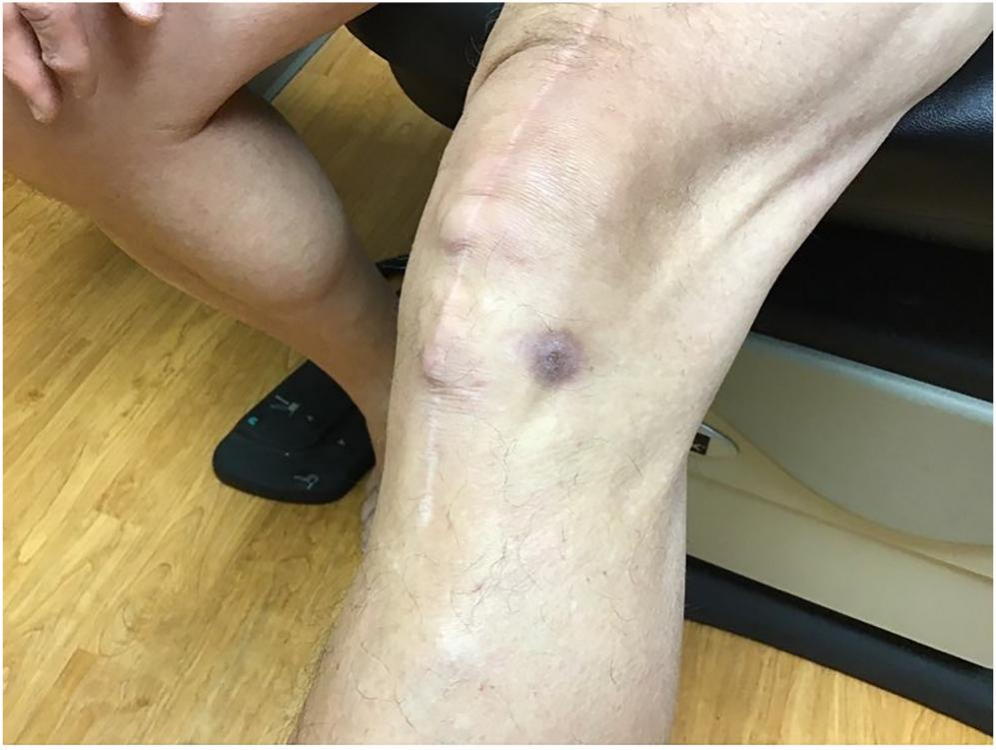
Post‐biopsy presentation with well‐healed linear scar at site of knee replacement

The metal in the patient's knee replacement was presumed the source of the cyst, and he was referred to orthopaedic surgery for replacement to prevent recurrence. Three months later, the patient was reassessed in the clinic. At this time, he described resolution of his knee pain, and physical exam showed a well‐healed linear scar at the site of replacement (Figure 6). The patient reported he had not seen his former orthopaedic surgeon but plans to do so to prevent recurrence.

## 3 DISCUSSION

In this case, the patient's history and physical exam findings, along with the dermatopathology evidence, reflect the characteristic pattern in patients suffering from MSCs. Prompt identification and subsequent removal can significantly improve patients' pain and ability to carry out daily activities. Therefore, it is of clinical significance to note the common features among patients presenting with MSCs.

As highlighted in other cases, this patient's MSC was related to prior surgical trauma.^1^ Other cases have also demonstrated a link between non‐surgical trauma and MSCs.^2^ Due to this common feature across patient presentations, MSCs should be a part of the differential when patients with history of trauma or surgery present with pain and a subcutaneous nodule.

Due to the association with surgical trauma, MSCs can be misdiagnosed as suture granulomas, which also present with pain and physically as subcutaneous nodules.^3^ However, dermatopathological analysis allows for differentiation between these two types of nodules. Whereas suture granulomas appear as epithelioid histiocytes and multinucleated giant cells surrounding a foreign body under the microscope,^4^ MSCs are visualised as papillary villous projections with synovial‐like lining, as noted above.

Metaplastic synovial cysts can cause a great deal of inconvenience for patients. Recognition of the condition can guide clinical management and hasten relief for patients. Our hope is that this case report will build upon the current knowledge regarding MSCs to improve diagnosis and management for these patients.

## CONFLICTS OF INTEREST

The authors have no conflicts of interest to declare.

## AUTHOR CONTRIBUTIONS

**Paayal S. Vora**: Writing – original draft (Equal). **Sonam Rama**: Conceptualization (Equal); Writing – review & editing (Equal). **Stephen Olsen**: Formal analysis (Equal).

## ETHICS STATEMENT

No IRB approval was required due to the nature of this case report and since it was not an actual study or collective patient review of charts or cases. Informed consent was obtained from the patient for publication of this case report and any accompanying images.

## Data Availability

The data that support the findings of this study are available from the corresponding author upon reasonable request.
